# Research on a Multimodel Fusion Diagnosis Method for Evaporation Ducts in the East China Sea

**DOI:** 10.3390/s23218786

**Published:** 2023-10-28

**Authors:** Cheng Zhang, Zhijin Qiu, Chen Fan, Guoqing Song, Bo Wang, Tong Hu, Jing Zou, Zhiqian Li, Sheng Wu

**Affiliations:** 1 Institute of Oceanographic Instrumentation, Qilu University of Technology (Shandong Academy of Sciences), Qingdao 266001, China; zhangchengl@hrbeu.edu.cn (C.Z.); fanchenqlu@163.com (C.F.); sgqgqing@163.com (G.S.); bob80.wang@hotmail.com (B.W.); tong.hu@qlu.edu.cn (T.H.); zoujing@qlu.edu.cn (J.Z.); lizhiqian@qlu.edu.cn (Z.L.); 2 Yantai Research Institute, Harbin Engineering University, Yantai 264000, China

**Keywords:** electromagnetic wave propagation, evaporation duct, multimodel fusion, Monin–Obukhov similarity theory, LIBSVM

## Abstract

Evaporation ducts are abnormal states of the atmosphere in the air–sea boundary layer that directly affect the propagation trajectory of electromagnetic (EM) waves. Therefore, an accurate diagnosis of the evaporation duct height (EDH) is important for studying the propagation trajectory of EM waves in evaporation ducts. Most evaporation duct models (EDMs) based on the Monin–Obukhov similarity theory are empirical methods. Different EDMs have different levels of environmental adaptability. Evaporation duct diagnosis methods based on machine learning methods only consider the mathematical relationship between data and do not explore the physical mechanism of evaporation ducts. To solve the above problems, this study observed the meteorological and hydrological parameters of the five layers of the low-altitude atmosphere in the East China Sea on board the research vessel Xiangyanghong 18 in April 2021 and obtained the atmospheric refractivity profile. An evaporation duct multimodel fusion diagnosis method (MMF) based on a library for support vector machines (LIBSVM) is proposed. First, based on the observed meteorological and hydrological data, the differences between the EDH diagnosis results of different EDMs and MMF were analyzed. When ASTD ≥ 0, the average errors of the diagnostic results of BYC, NPS, NWA, NRL, LKB, and MMF are 2.57 m, 2.92 m, 2.67 m, 3.27 m, 2.57 m, and 0.24 m, respectively. When ASTD < 0, the average errors are 2.95 m, 2.94 m, 2.98 m, 2.99 m, 2.97 m, and 0.41 m, respectively. Then, the EM wave path loss accuracy analysis was performed on the EDH diagnosis results of the NPS model and the MMF. When ASTD ≥ 0, the average path loss errors of the NPS model and MMF are 5.44 dB and 2.74 dB, respectively. When ASTD < 0, the average errors are 5.21 dB and 3.46 dB, respectively. The results show that the MMF is suitable for EDH diagnosis, and the diagnosis accuracy is higher than other models.

## 1. Introduction

The evaporation duct is an atmospheric layered structure that often appears at the air–sea interface. It is formed because the atmospheric humidity on the sea surface decreases rapidly with an increasing altitude under the marine atmospheric boundary layer (MABL), resulting in an abnormal refractive index of the atmosphere [1], a phenomenon that makes the distribution of the refractive index near the sea surface nonuniform. Evaporation ducts alter the propagation trajectory of electromagnetic (EM) waves through the atmosphere so that the EM waves can realize beyond-horizon detection and beyond-horizon communication in the duct layer and can obtain a detection blind zone [2,3,4]. Research on evaporation ducts can provide a basis for the detection of the marine radio wave environment and research on radio wave propagation characteristics and effectively avoid the EM wave path loss caused by the evaporation duct in the atmospheric environment [5,6]. By studying the diagnosis method of evaporation ducts, the evaporation duct height (EDH) can be obtained in real time, and the characteristics of evaporation ducts can be fully utilized to provide favorable conditions for long-distance detection, early warning, interception, communication, etc. In addition, relevant studies have shown that evaporation ducts usually appear in the range of 0–40 m height on the sea surface, and the occurrence probability of evaporation ducts in the South China Sea reaches 80%, which is extremely high [7,8,9]. Therefore, in practical applications, accurately diagnosing the EDH at sea is extremely important for improving the performance of the shipboard radar system in the evaporation duct.

At present, the main methods to obtain the EDH include direct detection, model diagnosis, machine learning methods, and so on. The direct detection method mainly uses detection equipment such as microwave refractometers, meteorological gradient towers, and low-altitude tethered balloons to obtain the observation data and obtain the modified refractivity profile (M profile). Then, it calculates the EDH according to the definition. However, this method is complicated to execute and has a high cost [10]. The model diagnosis method uses meteorological and hydrological data such as air temperature (AT), relative humidity (RH), air pressure (AP), wind speed (WS), and sea surface temperature (SST) at a certain height in a particular sea area to calculate the EDH [11]. Evaporation duct models (EDMs) mainly include the BYC [12,13], NPS [14,15,16], NWA [17], NRL [18], and LKB [19] models. EDMs are easy to use, but the parameterization methods of these models are based on empirical parameters obtained from observation experiments in specific sea areas, resulting in different diagnostic results of different EDMs under different meteorological conditions in different sea areas [20]. Machine learning methods can explore the internal relationship between data from a large amount of data to realize the diagnosis and prediction of data [21]. Therefore, machine learning methods have been applied to the diagnosis of EDH, and the expected effect has been obtained [22,23]. However, due to the lack of consideration of the physical mechanism of the evaporation duct, there are large deviations in the diagnosis results [24].

**Figure 1:**
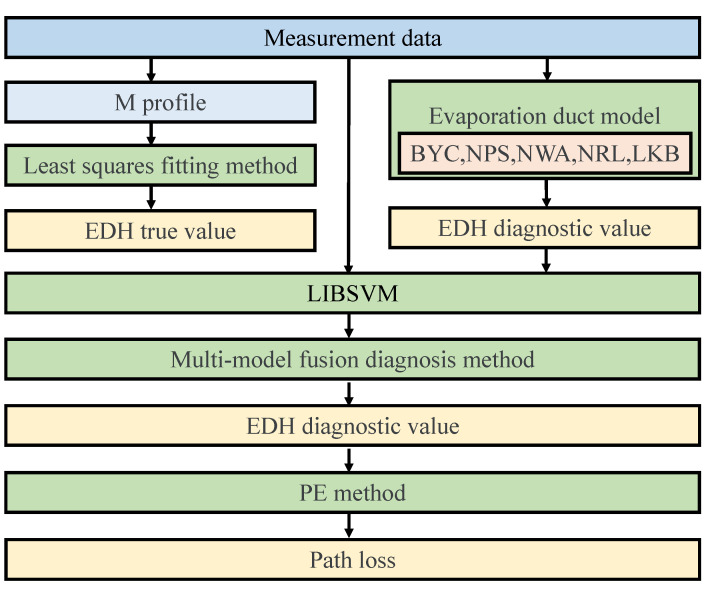
The flowchart for this study.

To solve the above problems, in this paper, based on a large amount of observation data in the East China Sea and a library for the support vector machines (LIBSVM) method, a multimodel fusion diagnosis method (MMF) for evaporation ducts is proposed to overcome these problems. Because the air–sea temperature difference (ASTD) is an important factor affecting the stability of the atmosphere, and different atmospheric stabilities have a greater impact on the diagnosis results of the evaporation duct model (EDM), therefore, the data are divided into two parts according to whether the ASTD is less than zero. The diagnostic results of the EDH were analyzed and verified, and the parabolic equation was used to analyze the EM wave path loss of the diagnosed EDH. A research flowchart of this study is shown in Figure 1. This study first obtains meteorological observation data at five layers through a scientific research vessel, calculates the M value of each layer, and then obtains the true profile of M through the least squares fitting method, and obtains the EDH true value through definition. The meteorological and hydrological observation data are used as the input of the EDMs to obtain the height diagnostic value of the EDM. Then, meteorological and hydrological observation data and EDH model diagnostic values are used as inputs to LIBSVM to construct a multimodel fusion diagnostic method. Finally, the accuracy of the EDH diagnostic value of the MMF is compared and analyzed. The EDH diagnostic value of the MMF is substituted into the parabolic equation to calculate the electromagnetic wave path loss and analyze its calculation accuracy.

## 2. Materials and Methods

### 2.1. Study Area and Data

**Figure 2:**
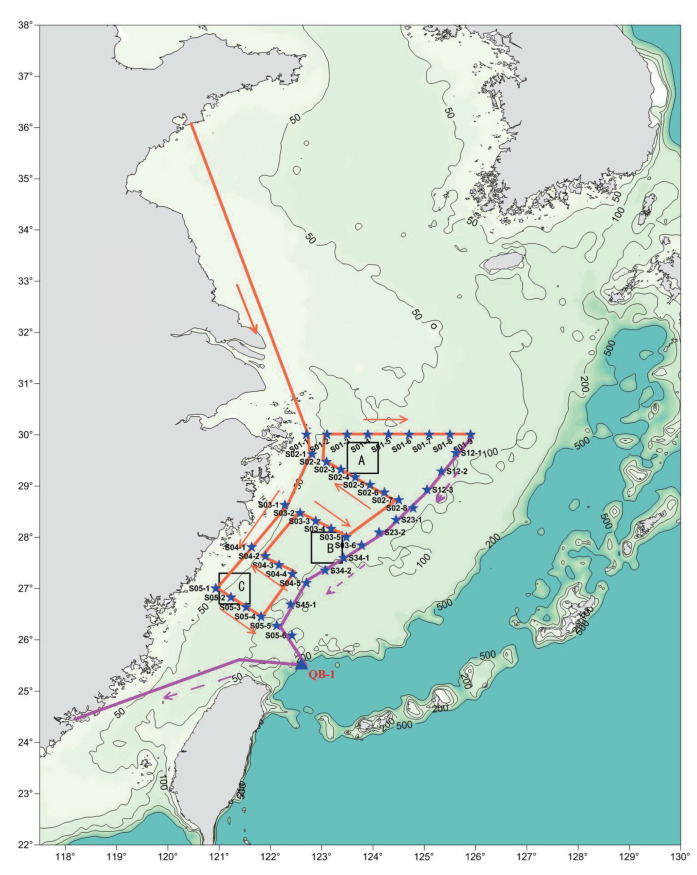
Voyage station and route map.

The East China Sea is a typical marginal sea in the western Pacific Ocean, surrounded by the Chinese Mainland, Taiwan Island of China, the Korean Peninsula, Japan’s Kyushu Island, and the Ryukyu Islands, and the sea weather conditions are very complicated [25]. To study the evaporation duct, 25°–32° N and 120°–127° E of the East China Sea was selected as the research area, and the meteorological and hydrological parameters were obtained by the shipborne gradient meteorological and hydrological sensors. During the “National Natural Science Foundation of China Open Research Cruise (Cruise No. NORC2021-02+NORC2021-301)” East China Sea Open Research Cruise, different temporal and spatial meteorological and hydrological gradient observation data in the East China Sea were obtained from 00:00:00 UTC+8 1 April 2021 to 23:59:59 UTC+8 23 April 2021. The voyage stations and routes are shown in Figure 2. Areas A, B, and C are the key research areas of this scientific research vessel. The points marked with ★ are the station points for this scientific research vessel’s operations. The orange line is the outbound route, and the purple line is the return route.

**Figure 3:**
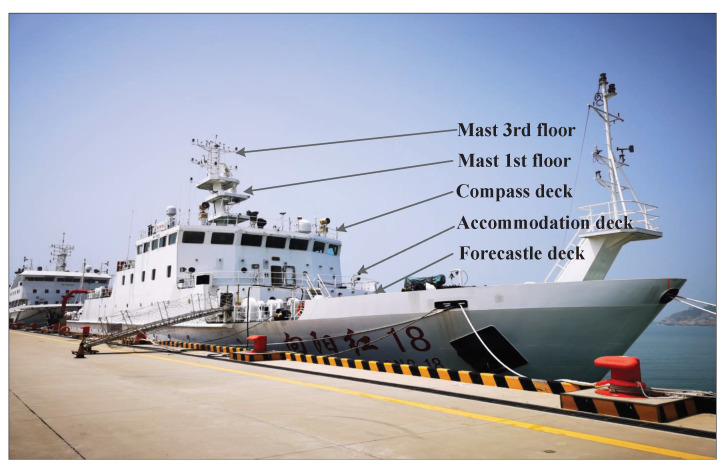
Schematic diagram of the sensor installation position of the R/V “XiangYangHong 18”.

To obtain meteorological and hydrological parameters at different heights, as shown in Figure 3, five layers of high-precision temperature and humidity sensors were installed on the bow deck (6 m), accommodation deck (8.3 m), compass deck (13.1 m), first layer mast (14.8 m), and third layer mast (22.3 m) of the R/V “Xiang YangHong 18” to achieve layered measurement of the AT, RH, and radio refractivity, N, at different heights. An Airmar WeatherStation was installed in the middle of the living deck to obtain observation data of the AT, RH, AP, and WS. A set of infrared temperature sensors was installed on the port and starboard to obtain the SST data. The collection frequency was set to 1 Hz [26]. The parameters of the meteorological and hydrological sensors used are shown in Table 1. After data preprocessing, a total of 22,771 sets of observation data were obtained, of which 9635 sets of data had ASTD ≥ 0, and 13,136 sets of data had ASTD < 0.

### 2.2. Methods

#### 2.2.1. Direct Detection Method

The propagation speed of EM waves in the atmosphere can be expressed by the refractive index *n*, *n* = $\frac{c}{v}$ (*c* is the propagation speed of EM waves in vacuum, that is, the speed of light; *v* is the propagation speed of EM waves in the air) [27,28]. Since the actual value of n is close to 1, it is inconvenient to calculate the propagation of EM waves in the atmosphere; it is usually represented by the radio refractivity *N*, *N* = (*n* − 1) $\times \;10^{6}$. *N* is closely related to meteorological parameters such as AP, AT, and RH. By obtaining the meteorological parameters of a certain altitude, Formula (1) can be used to calculate the *N* of the corresponding altitude. Due to this experiment, only the AP on the compass deck was obtained. When calculating *N* at other altitudes, the pressure height formula was used to convert AP [29], as shown in Formula (2).

(1) $$N=77.6\frac{AP}{AT}-5.6\frac{e}{AT}+3.73\times 10^{5}\frac{e}{AT^{2}},$$

where AT is the air temperature (k), AP is the air pressure (hPa), and *e* is the water vapor pressure (hPa).

(2) $$h-h_{0}=18410\left(1+at\right)log\frac{P_{0}}{P},$$

where the depth between atmospheric pressures *P* and $P_{0}$ is *h* − $h_{0}$ = Δh, and *t* is the average air temperature between *P* and $P_{0}$. *t* is given in degree centigrade (°C). a=1/273.

To express and explain the curvature of the Earth more conveniently, N is usually replaced by the modified refractivity M. The EDH can be obtained through the modified refractivity profile (M profile) [30,31]. The modified refractivity can be expressed by Formula (3).

(3) $$M=N+\frac{h}{R}\times 10^{6}=N+0.157h,$$

where $R=6.371\times 10^{6}$ is the mean radius of the earth, and *h* is the installation height of the sensor (m).

In this experiment, the meteorological observation data at the height of five layers obtained by the scientific research ship was used to determine the true value of the EDH. For the determination of EDH using multilayer meteorological observation data, generally, the least squares fitting method is used to obtain the corresponding modified refractivity M vertical profile. Its function expression is Formula (4).

(4) $$M=f_{0}z-f_{1}ln\left(z+z_{0}\right)+f_{2},$$

where $f_{0}$, $f_{1}$, and $f_{2}$ are undetermined coefficients, and *z* is the altitude above sea level; $z_{0}$ usually set to 0.001. dM/dz = 0 is the height corresponding to EDH.

#### 2.2.2. Evaporation Duct Model

The BYC, NPS, NWA, NRL, and LKB models use Monin–Obukhov similarity theory and the surface layer model theory to derive profiles of temperature and water vapor pressure that can be input Equation (1) to determine the refractivity profiles. The EDH is obtained according to the relationship between the EDH and the modified refractivity profile [32]. The water vapor pressure profile can be obtained from the specific humidity profile through Formula (5).

(5) $$e=\frac{qAP}{\varepsilon +(1-\varepsilon )q},$$

where *q* is the specific humidity (kg·kg${}^{-1}$), and ε is the ratio of the gas constant of dry air to water vapor.

The BYC, NPS, NWA, NRL, and LKB models are proposed for specific sea area observation experiments and have different parameterization methods, and the biggest difference between them is the use of different sea surface roughness methods and different stability functions ψ. Sea surface roughness methods include the dynamic roughness, temperature roughness, and humidity roughness. Stability functions include the wind function $\psi_{m}$ and the temperature-specific humidity function $\psi_{h}$; these functions are all functions of z/L (*z* is the measurement height, and *L* is the Monin–Obukhov length). The results of the stability functions of different models are almost consistent when z/L = 0; however, in other cases, there will be some differences. Usually, the stability functions of different models will be calculated separately according to the size of ζ = z/L.

a. BYC model

In the COARE2.5 flux algorithm, when ζ < 0, the $\psi_{m}$ and $\psi_{h}$ functions are Formulas (6) and (7).

(6) $$\psi_{m}=\frac{\psi_{uk}+\zeta \psi_{k}}{1+\zeta^{2}},$$

(7) $$\psi_{h}=\frac{\psi_{tk}+\zeta \psi_{k}}{1+\zeta^{2}},$$

where

$$\psi_{uk}=2\ln[\frac{1\;+\;z_{pu}}{p}]+\ln[\frac{1\;+\;z_{pu}^{2}}{2}]-2\arctan(z_{pu})+\frac{\pi }{2}$$

$$\psi_{k}=\mathrm{1.5}\ln[\frac{z_{pg}^{2}\;+\;z_{pg}\;+\;1}{3}]-\sqrt{3}\arctan[\frac{2z_{pg}\;+\;1}{\sqrt{3}}]+\frac{\pi }{\sqrt{3}}$$

$$\psi_{tk}=2\ln[\frac{1\;+\;z_{pt}}{2}]$$

$$z_{pu}=(1-16\zeta {)}^{0.25}$$

$$z_{pt}=(1-16\zeta ){}^{0.5}$$

$$z_{pg}=(1-12.87\zeta {)}^{\frac{1}{3}}.$$

When ζ ≥ 0, the $\psi_{m}$ and $\psi_{h}$ functions are Formula (8).

(8) $$\psi_{m}=\psi_{h}=-5\zeta .$$

b. NPS model

When ζ < 0, $\psi_{m}$ and $\psi_{h}$ function as the same as in the NPS model except that for $\psi_{m}$ is $z_{pg}$ = (1 − 10ζ)${}^{\frac{1}{3}}$ and for $\psi_{h}$ is $z_{pg}$ = (1 − 34ζ)${}^{\frac{1}{3}}$.

When ζ ≥ 0, the $\psi_{m}$ and $\psi_{h}$ functions are Formulas (9) and (10).

(9) $$\psi_{m}=-\zeta -\frac{2}{3}\left[\zeta -\frac{5}{0.35}\right]exp\left(-0.35\zeta \right)-\frac{2}{3}\cdot \frac{5}{0.35},$$

(10) $$\psi_{h}=1-{\left[1+\frac{2}{3}\zeta \right]}^{1.5}-\frac{2}{3}\left[\zeta -\frac{5}{0.35}\right]exp\left(-0.35\zeta \right)-\frac{2}{3}\cdot \frac{5}{0.35}.$$

c. NWA model

When ζ < 0, the $\psi_{m}$ and $\psi_{h}$ functions are Formulas (11) and (12).

(11) $$\psi_{m}=2ln\left[\frac{1+z_{pu}}{2}\right]+ln\left[\frac{1+z_{pu}^{2}}{2}\right]-2arctan\left(z_{pu}\right)+\frac{\pi }{2},$$

(12) $$\psi_{h}=2ln\left[\frac{1+z_{pu}^{2}}{2}\right],$$

where


$$z_{pu}=(1-16\zeta {)}^{0.25}.$$

When ζ ≥ 0, the $\psi_{m}$ and $\psi_{h}$ functions are Formula (13).

(13) $$\psi_{m}=\psi_{h}=-6ln\left(1+\zeta \right).$$

d. NRL model

When ζ < 0, the NRL model uses the same $\psi_{m}$ and $\psi_{h}$ functions as the NWA model.

When ζ ≥ 0, the $\psi_{m}$ and $\psi_{h}$ functions are Formula (14).

(14) $$\psi_{m}=\psi_{h}=-7\zeta .$$

e. LKB model

When ζ < 0, the $\psi_{m}$ and $\psi_{h}$ functions are the same as in the NRL model except that $z_{pu}$ = (1 + 16ζ)${}^{0.25}$ [19].

When ζ ≥ 0, the LKB model uses the same $\psi_{m}$ and $\psi_{h}$ functions as the NRL model.

#### 2.2.3. Multimodel Fusion Diagnosis Method

**Figure 4:**
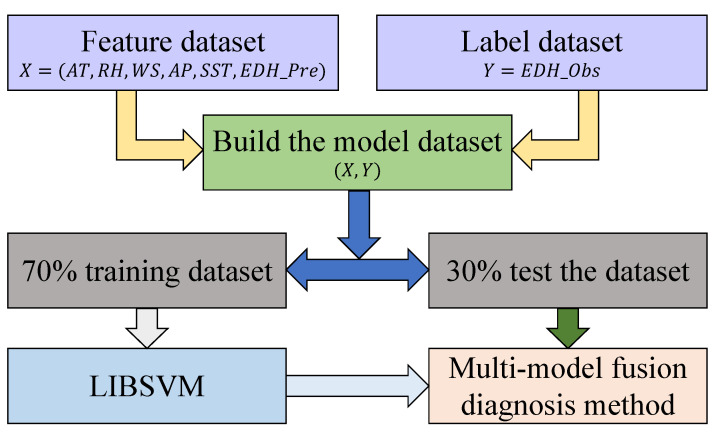
Flow chart of the MMF.

As shown in Figure 4, to improve the applicability and accuracy of the EDM, taking into account the physical mechanism of an evaporation duct and the advantages of machine learning methods, the MMF was constructed. The MMF adopted LIBSVM to carry out the data fusion of meteorological and hydrological parameters and the diagnosis results of the BYC, NPS, NWA, NRL, and LKB models.

First, the meteorological and hydrological data were preprocessed to eliminate invalid data, obtain valid AT, WS, RH, AP, and SST data, and use them as the inputs to obtain the EDH dataset EDH_Pre of the BYC, NPS, NWA, NRL and LKB models. The processed meteorological and hydrological data were merged with EDH_Pre as a feature dataset *X* = (AT,RH,WS,AP,SST,EDH_Pre), and the observed EDH *Y* = EDH_Obs was used as the label dataset. According to whether the ASTD was less than zero, the dataset was divided into two parts, and the fusion modeling training was carried out, respectively. The dataset was normalized so that the preprocessed data were limited to a certain range, thereby eliminating the adverse effects caused by different data dimensions. The kernel function chooses the radial basis function, because the kernel function can nonlinearly map the data to a high-dimensional space and can also deal with the nonlinear relationship between features and their attributes. Seventy percent of the data were randomly obtained as the training dataset, and the remaining 30% of the dataset were the testing dataset. The cross-validation method was used to select the best penalty factor c and kernel function parameter g for the training. Then, the LIBSVM method was used to carry out the fusion method modeling training, and the MMF model was obtained. Finally, the testing dataset was used to test, compare, and improve the diagnosis results of the evaporation duct.

## 3. Results and Discussion

**Figure 5:**
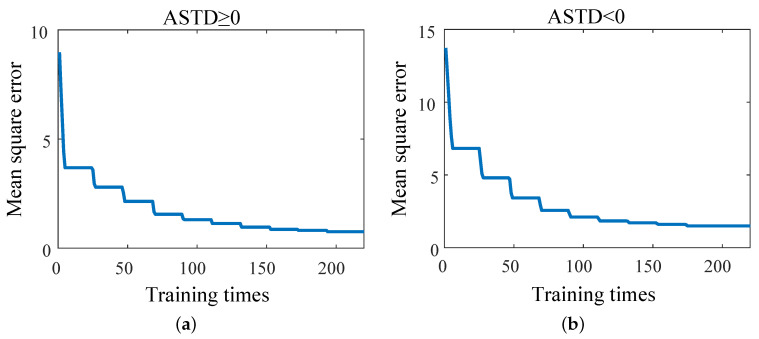
MSE of the evaporation duct diagnostic results and true values corresponding to different c and g during the cross-validation process: (**a**) ASTD ≥ 0; (**b**) ASTD < 0.

**Figure 6:**
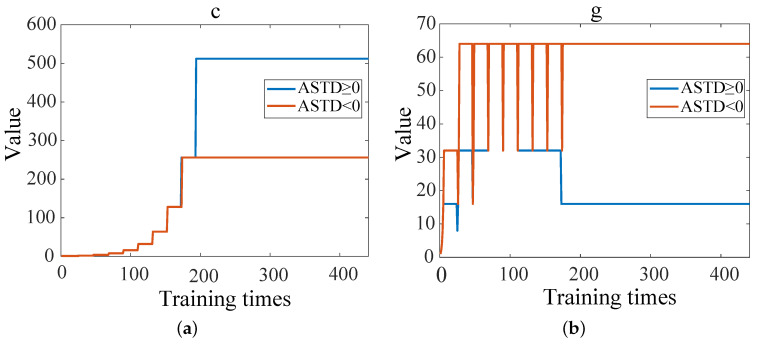
The change curves of optimal c and g during training: (**a**) the change of curve of optimal c; (**b**) the change of curve of optimal g.

**Figure 7:**
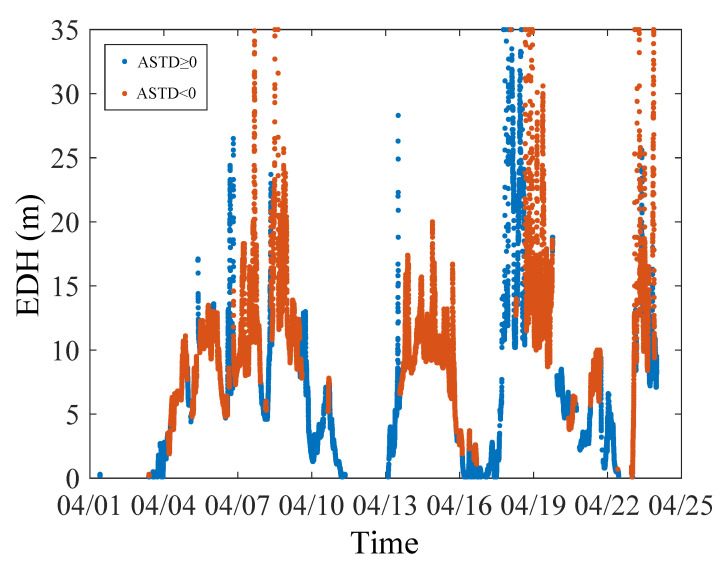
Timeline of true values of EDH.

Figure 5 shows the mean square error (MSE) between the diagnostic results of the EDH and the true value corresponding to different c and g when the cross-validation process is plotted. When ASTD ≥ 0, the MSE is 0.75 after 194 trainings; when ASTD < 0, the MSE is 1.49 after 175 trainings. The change curves of the optimal c and g during the training process are shown in Figure 6. The optimal c and g parameters are found in multiple trainings through the cross-validation method, as shown in Table 2. Figure 7 shows the data when the EDH is ASTD ≥ 0 and ASTD < 0.

**Figure 8:**
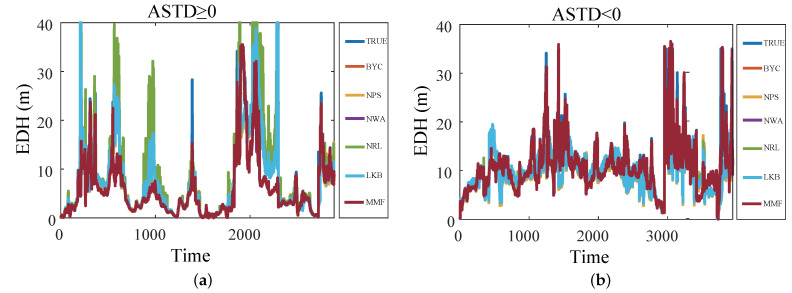
The diagnosis results of the EDH by the five EDMs and MMF: (**a**) ASTD ≥ 0; (**b**) ASTD < 0.

Figure 8 shows the comparisons of the diagnosis results of the EDH. In order to evaluate the comprehensive performance of the BYC, NPS, NWA, NRL, LKB, and MMF models, the diagnosis results of the six diagnosis models are compared with the true values. Regardless of whether ASTD ≥ 0 or ASTD < 0, the diagnostic value of the EDH of the MMF is closer to the true value than the other five models. In addition, the accuracy and correlation of the diagnosis results of the MMF are better than those of the five EDMs. This shows that the MMF can be applied to the optimization research of evaporation duct diagnosis.

**Figure 9:**
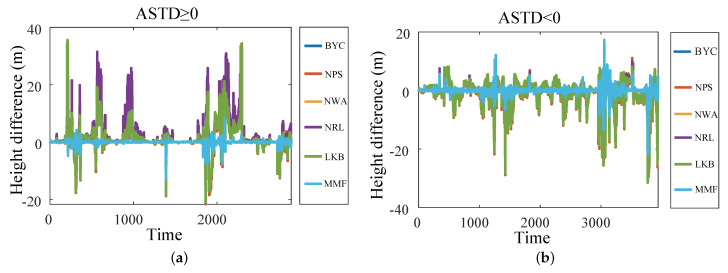
The difference between the diagnosis results of each model and the true value: (**a**) ASTD ≥ 0; (**b**) ASTD < 0.

To analyze the diagnosis results of the BYC, NPS, NWA, NRL, LKB, and MMF more specifically, the difference between the diagnosis results of each model and the true value was further compared and analyzed. As shown in Figure 9, when ASTD ≥ 0, the diagnostic results of the MMF were almost consistent with the true value; the deviation between it and the true value was the smallest, and the maximum difference was only 13.01 m. The difference between the diagnostic results of the NRL model, the LKB model, and the true value was the largest, and the maximum difference reached 35.60 m. The average differences between the diagnostic results of the EDM and the true value were 2.57 m, 2.92 m, 2.67 m, 3.27 m, 2.57 m, and 0.24 m, respectively. When ASTD < 0, the maximum difference of the diagnosis results of the BYC, NPS, NWA, NRL, and LKB models reached more than 31 m, while the maximum difference in the MMF was only 21.74 m. The average differences between the diagnostic results of the EDM and the true values were 2.95 m, 2.94 m, 2.98 m, 2.99 m, 2.97 m, and 0.41 m, respectively. No matter whether ASTD ≥ 0 or ASTD < 0, the deviation in the diagnostic results of the MMF was smaller than that of the BYC, NPS, NWA, NRL, and LKB models, which shows that the MMF has a better diagnostic accuracy.

To quantitatively evaluate the results, as shown in Table 3, the root mean square error (RMSE) and Pearson correlation coefficient (PCC) were used to analyze the diagnostic results. First, RMSE analysis was carried out. When ASTD ≥ 0, the RMSE of the NRL model diagnosis was the largest, reaching 6.17 m, and the RMSE of diagnosis by MMF was the smallest, which was 0.72 m. When ASTD < 0, the RMSEs of the BYC, NPS, NWA, NRL, and LKB models diagnostic results were basically consistent, and all were approximately 4.8 m; the RMSE of the diagnostic result of the MMF was 1.17 m, which was much smaller than the other model diagnostic result. This shows that the diagnostic accuracy of the MMF was significantly improved. Then, the PCC analysis showed that when ASTD ≥ 0, the correlation coefficient between the diagnosis results of the NRL model and the true value was the smallest, with only a value of 0.71; the PCC between the diagnosis results of the MMF and the true value was the largest, reaching 0.99. When ASTD < 0, the PCC of the diagnosis results of the BYC, NPS, NWA, NRL, and LKB models was basically consistent with the true value, all of which were approximately 0.51; the PCC of the MMF diagnosis result and the true value was 0.97. This shows that the correlation of MMF was better than that of the other models.

**Figure 10:**
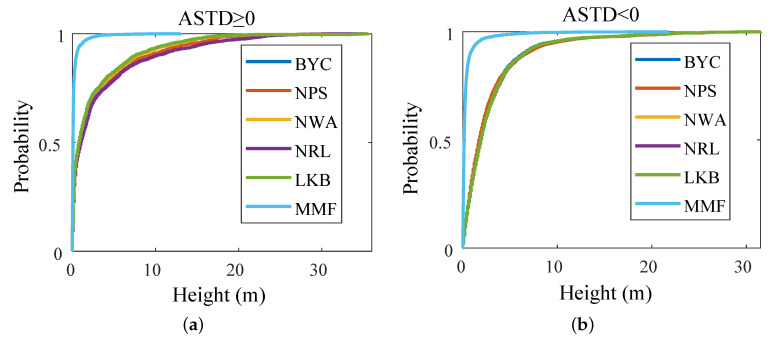
CDF of the difference between the diagnostic EDH and the true value: (**a**) ASTD ≥ 0; (**b**) ASTD < 0.

Figure 10 is the cumulative distribution function (CDF) of the difference between the diagnostic EDH and the true value obtained by the model diagnosis and the MMF of the meteorological and hydrological parameters of the voyage. When ASTD ≥ 0, the probability that the error of the diagnosis result of the MMF was within 0.5 m was above 0.9, while that of the other models was below 0.5; when ASTD < 0, the probability that the error of the diagnosis result of the MMF was within 0.8 m was above 0.9, while that of other models was below 0.3. This shows that the diagnosis results of the MMF are more stable.

**Figure 11:**
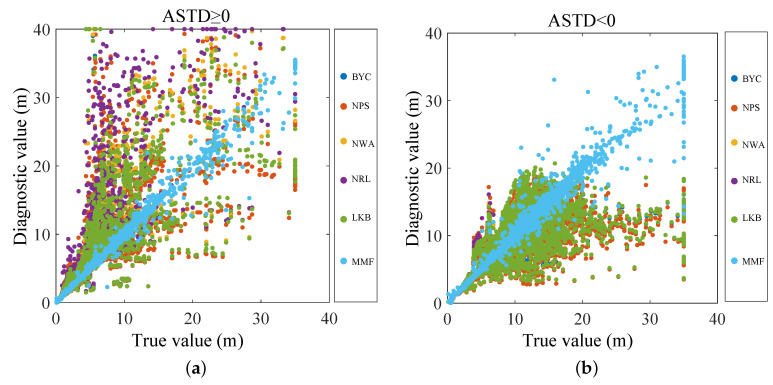
Comparison of the diagnostic values with the true values: (**a**) ASTD ≥ 0; (**b**) ASTD < 0.

As shown in Figure 11, the diagnostic results of the evaporation duct were compared with the true values using the scatter plot. The diagnosis results and true values of the MMF were almost on a straight line in the middle, and the diagnosis accuracy was much better than those of the other five EDMs.

To further study the performance of the MMF under electromagnetic wave propagation when the radar operating frequency was 10 GHz, the antenna height was 10 m, the beam width was 3°, the elevation angle was 0°, and horizontal polarization was used for the true value of the evaporation duct, the diagnosis results of the NPS model, and the MMF. Moreover, the parabolic equation (PE) was used to calculate the path loss of the electromagnetic wave and analyze the results.

**Figure 12:**
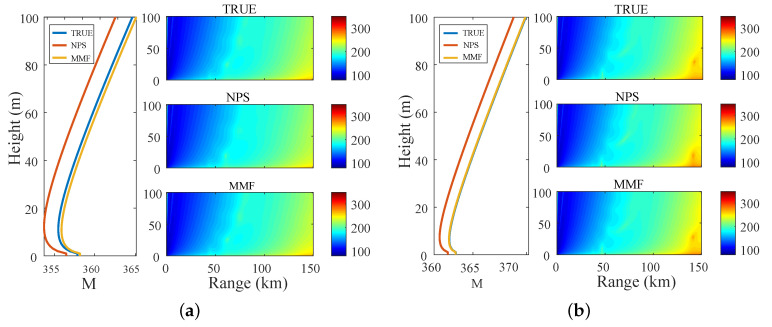
Atmospheric corrected refractive index profile and corresponding path loss distribution: (**a**) ASTD > 0; (**b**) ASTD < 0.

Figure 12 shows the calculation results of the M profile and the corresponding EM wave path loss spatial variation where (a) is the calculation result corresponding to the ASTD > 0 at 19:32:00 UTC+8 on 17 April 2021, and (b) is the calculation result corresponding to the ASTD < 0 at 16:20:00 UTC+8 on 4 April 2021. When ASTD > 0, the diagnostic results of the NPS model and the MMF were basically consistent with the spatial variation of the EM wave path loss of the true value of the EDH. When ASTD < 0, the spatial variation of the EM wave path loss was significantly different. Meanwhile, the M profile and the EM wave path loss of the MMF were closer to the true value than the NPS model.

**Figure 13:**
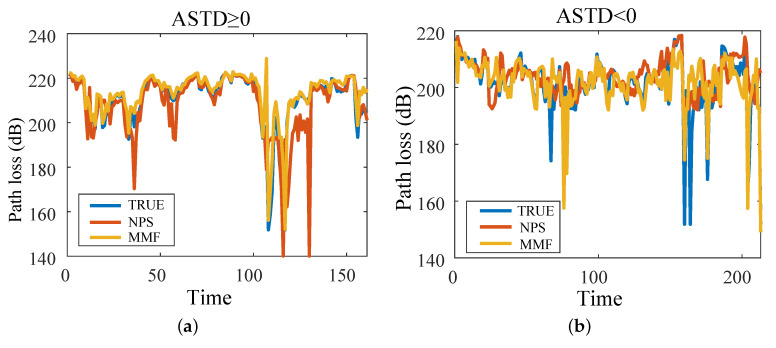
Path loss calculated at 100 km range and 10 m height: (**a**) ASTD ≥ 0; (**b**) ASTD < 0.

**Figure 14:**
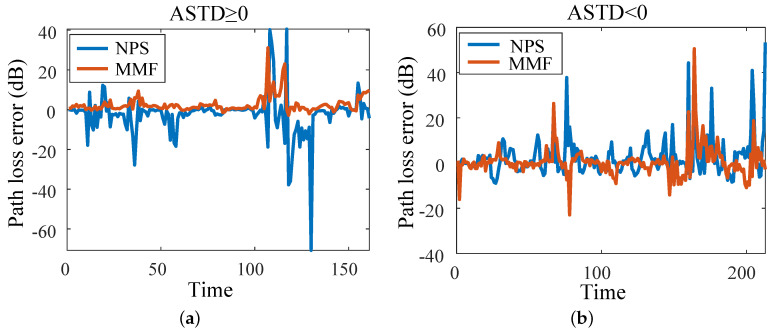
Path loss error between NPS, MMF, and the true values: (**a**) ASTD ≥ 0; (**b**) ASTD < 0.

Figure 13 shows the EM wave path loss value at a propagation distance of 100 km and a height of 10 m (161 h of data when ASTD ≥ 0, and 213 h of data when ASTD < 0). The path loss errors of the NPS model and the MMF and the true value are shown in Figure 14. When ASTD ≥ 0, the average path loss errors of the NPS model and the MMF were 5.44 dB and 2.74 dB, and the RMSEs were 10.95 dB and 4.84 dB, respectively. When ASTD < 0, the average path loss errors of the NPS model and the MMF were 5.21 dB and 3.46 dB, and the RMSEs were 9.17 dB and 6.48 dB, respectively. The average error and RMSE of the path loss of the MMF were smaller than those of the NPS model, indicating that the path loss accuracy of the MMF was better than that of the NPS model. Moreover it is suitable for research on the EM wave path loss.

## 4. Conclusions

The parameterization methods of the BYC, NPS, NWA, NRL, and LKB models are all based on empirical parameters obtained from observation experiments in specific sea areas. Therefore, under different maritime meteorological conditions, the diagnostic results of different EDMs often have some differences. While machine learning methods can explore the hidden laws between data, they often ignore the physical mechanism of the evaporation duct. Therefore, this study proposes an MMF for the evaporation duct based on LIBSVM. The MMF considers not only the data association characteristics but also the physical mechanism of the evaporation duct. Thus, it has a strong diagnosis ability and is very significant for researching EM wave propagation characteristics at sea.

This paper uses the meteorological and hydrological observation data of the East China Sea voyage to analyze the diagnostic errors between five EDMs and the MMF and the evaporation duct true value under different meteorological and hydrological conditions. When ASTD ≥ 0, the average error of the MMF is only 0.24 m, while other models are greater than 2.5 m; The RMSE of the MMF is 0.72 m, while other models reach a maximum of 6.17 m; The PCC of the MMF is 0.99, which is much higher than other models. When ASTD < 0, the average error of the MMF is only 0.41 m, while other models are greater than 2.9 m. The RMSE of the MMF is 1.17 m, which is much smaller than other models. The PCC of the MMF is 0.97, which is much larger than other models. The results show that regardless of whether ASTD ≥ 0 or ASTD < 0, the diagnostic error of the MMF was smaller than the other models and had better correlation. Further, the NPS model and the MMF diagnosis results were used as the input to the PE, and the influence of the NPS model and the MMF diagnosis accuracy on the EM wave path loss was analyzed. The average error and RMSE of the path loss of the MMF were smaller than those of the NPS model. The results show that the diagnosis accuracy of the EM wave path loss of the MMF is higher than that of the NPS model.

The above analysis used the data of the East China Sea in April, but whether the MMF is suitable for other months or other sea areas remains to be studied. We will next obtain data from other months in the East China Sea for analysis and further verify the generalization ability of the MMF with observation data from other sea areas. Since it is difficult to observe data at sea, and scientific research ships are easily affected by large waves in the deep sea, in the following research, offshore observation towers, buoys, and other platforms will be used to obtain more observation data and further analyze the conclusions. This article uses LIBSVM to fuse data. In subsequent work, other machine learning methods will be used to fuse data, and the advantages and disadvantages of using different machine learning methods for fusion will be analyzed.

Combined with the existing observation data, we will next analyze the reasons why the MMF has large deviations at certain times to further improve the MMF and the accuracy of the MMF.

## Figures and Tables

**Table 1:** Meteorological and hydrological sensor parameters.

Sensor	Type	Data	Specification	Location
HUMICAP	Vaisala HMP155	Relative humidity	RH:	Mast 3rd level (22.3 m)
			15–25 °C: ±1% RH (0–90% RH)	Mast 1st level (14.8 m)
			−20–40 °C: ±1.7% RH (90–100% RH)	Accommodation deck (8.3 m)
		Air temperature	AT: ±(0.055–0.0057 × AT °C)	Accommodation deck (8.3 m)
				Forecastle deck (6 m)
Barometric pressure	Young 61302L	Air pressure	±0.2 hPa (25 °C), ±0.3 hPa (−40–60 °C)	Compass deck (13.1 m)
Weather station	Airmar 150WXS	Wind speed	5% (10 m/s)	Compass deck (13.1 m)
Infrared thermometers	Optris CTLT20	Sea surface temperature	±1 °C	Compass deck (13.1 m)

**Table 2:** The optimal parameters of c and g obtained by training.

Condition	Parameter	Data
ASTD ≥ 0	c	512
	g	16
ASTD < 0	c	256
	g	64

**Table 3:** The RMSE and PCC of the MMF and the five models.

Parameter	Model	Atmospheric Condition	
		ASTD ≥ 0	ASTD < 0
RMSE	BYC	4.93	4.75
	NPS	5.48	4.86
	NWA	5.04	4.77
	NRL	6.17	4.78
	LKB	4.93	4.77
	MMF	0.72	1.17
PCC	BYC	0.76	0.51
	NPS	0.72	0.51
	NWA	0.76	0.50
	NRL	0.71	0.50
	LKB	0.76	0.50
	MMF	0.99	0.97

## Data Availability

The data presented in this study are available on request from the corresponding author.
